# Noninvasive Assessment of Exosome Pharmacokinetics In Vivo: A Review

**DOI:** 10.3390/pharmaceutics11120649

**Published:** 2019-12-03

**Authors:** Do Hee Kim, Vinoth Kumar Kothandan, Hye Won Kim, Ki Seung Kim, Ji Young Kim, Hyeon Jin Cho, Yong-kyu Lee, Dong-Eun Lee, Seung Rim Hwang

**Affiliations:** 1 College of Pharmacy, Chosun University, 309 Pilmun-daero, Dong-gu, Gwangju 61452, Korea; gogoupzz@gmail.com (D.H.K.); khwon0628@gmail.com (H.W.K.); jikol5216@naver.com (K.S.K.); cacaco72@naver.com (J.Y.K.); chj347@naver.com (H.J.C.); 2 Department of Biomedical Sciences, Graduate School, Chosun University, 309 Pilmun-daero, Dong-gu, Gwangju 61452, Korea; vino2787@ymail.com; 3 Department of Chemical and Biological Engineering, Korea National University of Transportation, Chungju, Chungbuk 27469, Korea; leeyk@ut.ac.kr; 4 Advanced Radiation Technology Institute, Korea Atomic Energy Research Institute, Jeongeup, Jeonbuk 56212, Korea; delee@kaeri.re.kr

**Keywords:** exosome, pharmacokinetic, imaging, labelling, distribution

## Abstract

Exosomes, intraluminal vesicles that contain informative DNA, RNA, proteins, and lipid membranes derived from the original donor cells, have recently been introduced to therapy and diagnosis. With their emergence as an alternative to cell therapy and having undergone clinical trials, proper analytical standards for evaluating their pharmacokinetics must now be established. Molecular imaging techniques such as fluorescence imaging, magnetic resonance imaging, and positron emission tomography (PET) are helpful to visualizing the absorption, distribution, metabolism, and excretion of exosomes. After exosomes labelled with a fluorescer or radioisotope are administered in vivo, they are differentially distributed according to the characteristics of each tissue or lesion, and real-time biodistribution of exosomes can be noninvasively monitored. Quantitative analysis of exosome concentration in biological fluid or tissue samples is also needed for the clinical application and industrialization of exosomes. In this review, we will discuss recent pharmacokinetic applications to exosomes, including labelling methods for in vivo imaging and analytical methods for quantifying exosomes, which will be helpful for evaluating pharmacokinetics of exosomes and improving exosome development and therapy.

## 1. Introduction

Exosomes are intraluminal vesicles released by cells when multivesicular endosomes fuse with the cellular plasma membranes [1,2]. They contain lipid membranes, nucleic acids, and proteins derived from the original donor cells, circulate in body fluids, and deliver their cargo to the recipient cells, resulting in intercellular communication [3,4]. As exosomes contain informational cargo from the original donor cells and exist in tissues as well as bodily fluids including blood, urine, and saliva, they possess diagnostic potential [5,6,7]. Researchers have been using analytical tools such as mass spectrometry and proteomic profiling to find specific exosomal biomarkers [8,9].

In addition, exosomes have shown their potential as an alternative to cell therapy, supported by data indicating the therapeutic feasibility and safety of exosomes in preclinical and clinical studies. Dendritic cell (DC)-derived exosomes containing major histocompatibility complex/peptide and activating T cell immune response were tested as vaccines against metastatic melanoma and non-small-cell lung cancer in clinical trials [10,11,12]. Combinations of exosomes isolated from ascites containing tumor antigens with immune-stimulatory adjuvants were also investigated in clinical trials involving advanced colorectal cancer patients [13,14]. Recently, stem-cell-derived exosomes have been developed to target cardiovascular, diabetic, graft-versus-host, neurological, and orthopedic diseases [15,16,17]. Plant-derived exosomes have also undergone clinical tests for delivery of curcumin [18,19].

For efficient preclinical and clinical tests, pharmacokinetic properties need to be examined from the early stage of exosome-based drug development. In the preclinical phase, exosome-based drug candidates are administered to laboratory animals by the expected clinical route of administration, and their absorption, distribution, metabolism, and excretion (ADME) can be checked by using fluorescence labelling or radiolabeling of exosomes [20]. Methods for quantifying metabolites in blood, tissue, and urine also should be established [21]. As shown in Table 1, calculation of pharmacokinetic parameters such as volume of distribution, clearance, and absorption or elimination rate constant will be helpful to predict the toxicity and efficacy of exosome-based drug candidates [22]. Exosome concentration in tissues over time depends on exosome concentration in blood, and the time course of exosome concentration in blood is affected by the dose and route of administration [23,24].

In this review, we will discuss the pharmacokinetic approach to exosomes, labelling methods for in vivo imaging, and analytical methods for quantifying exosomes. A review of recent applications of pharmacokinetics will be helpful for evaluating pharmacokinetics of exosomes and improving exosome development and therapy.

## 2. ADME of Exosomes

Membrane transport plays an important role in absorption, secretion, or excretion of exosomes. In in vitro transport studies, milk exosomes penetrate Caco-2 cells and rat small intestinal epithelial cells by endocytosis, with the help of glycoproteins on the surface of exosomes and epithelial cells [25]. There are various views on the detailed mechanism of exosome endocytosis [27]. Uptake of exosomes into PC12 cells is inhibited by potassium ion depletion for blocking clathrin-coated pits, implying receptor-mediated endocytosis in cellular uptake of exosomes [28]. Oligodendrocyte-derived exosomes suspended in extracellular fluid may be internalized into microglia through macropinocytosis that does not require binding to the specific receptor [29]. Phagocytic cells, such as macrophages, engulf exosomes via PI3-kinase-dependent phagocytosis, and then the exosome-containing phagosomes fuse with lysosomes [30]. Moreover, it was reported that exosomes penetrate the nasal mucosa through the olfactory pathway and directly enter the brain by bypassing the blood–brain barrier [31,32]. Active transport of human ovarian carcinoma-derived exosomes across lymphatic endothelial cells has also been shown in vitro, where the lymphatic transport was supported by the presence of exosomes in the lymph nodes after intradermal injection in mice [33].

After being absorbed at the administration site and transferred to the systemic circulation, exosomes pass blood–tissue barriers and arrive in each tissue [34]. Exosomes injected into the veins of mice are mainly distributed in the organs with a mononuclear phagocyte system (MPS) such as the liver, spleen, lungs, and kidneys, so macrophage-derived exosomes can improve targeting of MPS organs [26,35,36,37]. In a Parkinson’s disease mouse model, distribution of exosomes to the brain was due to the interaction with cerebral microvascular endothelial cells that were enhanced in the inflamed brain [26]. Tissue distribution of exosomes is related to its therapeutic efficacy and safety issues, such as accumulation in tissues and side effects [38]. When unmodified exosomes were intravenously injected into 4T1-bearing mice, they mainly accumulated in the liver and spleen, with limited accumulation in the lungs and kidneys as well as little accumulation in the tumor tissue [39]. Thus, there is a need for exosomes to be excreted after staying at the target site for the required time and for minimizing exosome transition to other off-target organs. In recent years, research on exosomes with targeting ligands has been actively conducted [40,41]. To realize mouse brain-targeting small interfering RNA cargo, exosomes were isolated from DCs expressing neuron-specific rabies viral glycoprotein peptide cloned into an exosome membrane protein [40]. Exosomes can be modified to allow for specific binding to breast cancer cells expressing an epidermal growth factor receptor by plasmid transfection [41].

After exosomes that reach the site of action are internalized by the recipient cells, their cargo should work at the cytosolic space without being trapped in endosomes and degraded by acidic pH or hydrolases [42]. Tumor-derived exosomes modified with a pH-sensitive fusogenic peptide showed improved cargo delivery to the cytosol and class I tumor antigen presentation [43]. Folate-displaying exosomes have been reported to deliver their cargo siRNA to the cytosol through membrane fusion, and show better gene silencing efficiency than that of folate-conjugated siRNA without exosomes [44].

**Figure 1:**
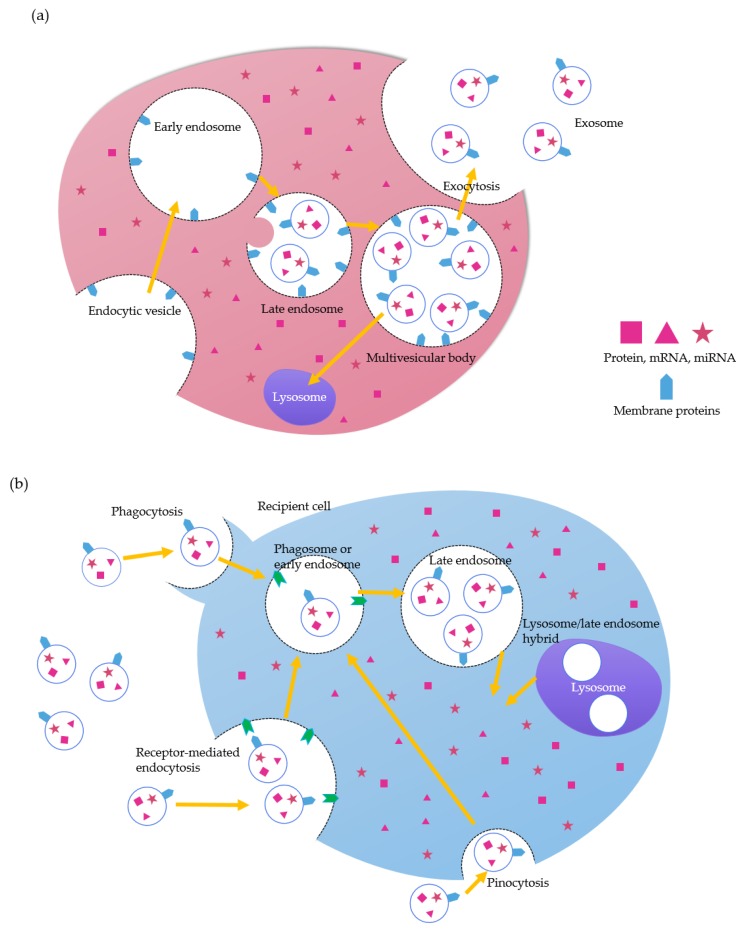
Production and cellular uptake of exosomes. (**a**) Production of exosomes. (**b**) Cellular uptake and endocytic pathway of exosomes.

Absorbed exosomes will be eliminated from the body through metabolism and excretion. There are several reports on analysis of metabolites from exosomal proteins, RNAs, and lipid membranes. The exosome metabolome contains nucleotides, nucleosides, carboxylic acids, amino acids, sugars, cyclic alcohols, carnitines, vitamins, and lipids (fatty acids, glycerolipids, glycerophospholipids, sphingolipids, sterol lipids, and prenol lipids) [45,46,47]. After cellular uptake, the cargo released from exosomes will undergo the endocytic pathway (Figure 1) consisting of early endosomes, late endosomes, and lysosomes, resulting in breaking down or recycling [48,49].

Among other organs, the kidneys, liver, and spleen, where fixed macrophages stay, have been reported to be most closely associated with clearance of exosomes. This is supported by results that pretreatment of clodronate to deplete Kupffer cells in the liver and red pulp macrophages in the spleen significantly reduce clearance of exosomes in mice [23].

## 3. Recent Applications of Pharmacokinetics

**Scheme 1:**
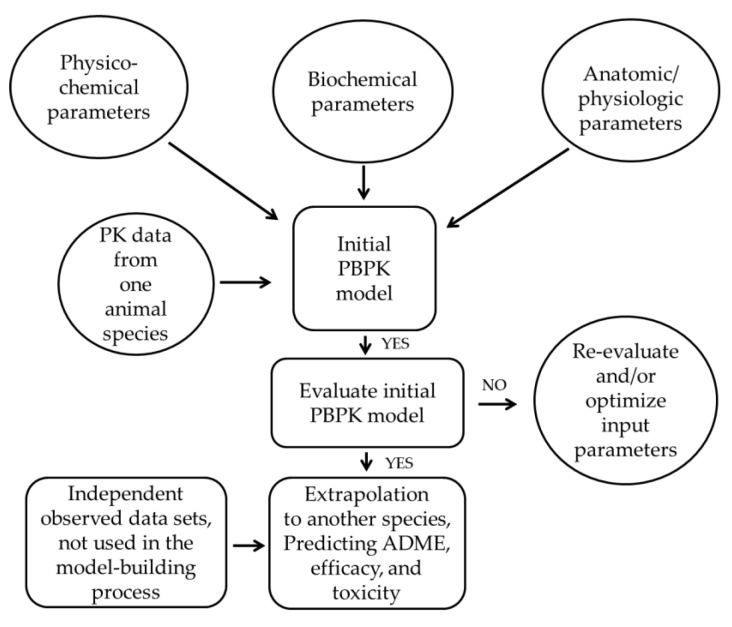
Flow diagram of a physiologically based pharmacokinetic (PBPK) model.

In addition to existing approaches based on compartment models, there have been reports of predicting ADME, efficacy, and toxicity of drugs by applying physiologically based pharmacokinetic (PBPK) modeling [50]. The PBPK model should consist of predefined organs or tissues in connection with physiological, physicochemical, and biochemical parameters, such as blood flow, organ volume, and clearance, in order to calculate drug concentration in blood and concentration changes in each organ or tissue according to time [51]. Using PBPK models, pharmacokinetic data can be extrapolated from one animal species to another animal species or humans (Scheme 1), and we can determine the safety levels of the drug [52]. Recently, the PBPK model and pharmacokinetic simulation have been applied to liposomal drug development, which would contribute to evaluation of exosomal disposition and the physiological impact of exosomal cargo [53].

Analytical methods for exosomes circulating in body fluids and accumulated in each tissue or organ also need to be developed and validated for phenotypic screening and optimizing individual dosage regimens, because of variations in ADME of exosomes between patients due to individual genetic variations [54,55]. Molecular imaging techniques such as fluorescence imaging, magnetic resonance imaging, single photon emission computed tomography (SPECT), and positron emission tomography (PET) enable us to observe ADME of exosomes [56].

## 4. Methods of Exosome Labeling for Pharmacokinetic Study

### 4.1. Fluorescent Labeling of Exosomes

Fluorescent labeling is useful for whole body imaging of exosomes, because we can detect fluorescence emission in response to the excitation light of a specific wavelength time-efficiently and noninvasively [57]. Near-infrared (NIR) dyes such as indocarbocyanine analogs have been used for tracking lipid membrane of exosomes in mice with minimal autofluorescence of biological tissue and high tissue penetration in a spectral range of 700–900 nm [58,59]. However, lipid labeling with fluorescent dyes may lead to inaccurate measurement of the half-life of labeled exosomes due to the longer fluorescent signal of the free lipophilic dye released from exosomes in vivo. Quenching of fluorescence caused by aggregated dyes should be carefully validated, too [60].

### 4.2. Luminescent Labeling of Exosomes

Luminescent labeling and bioluminescence imaging of exosomes using the enzyme reaction also have been reported. Exosomes could be labeled with a fusion protein of *Gaussia* luciferase and lactadherin (gLuc-lactadherin) by transfecting the original donor cells with a plasmid expressing gLuc-lactadherin, and tissue distribution of exosomes following intravenous injection in mice was evaluated through bioluminescence detection [35]. Combining *Gaussia* luciferase with a biotin acceptor domain created a multimodal imaging reporter with accurate spatiotemporal resolution for labeling exosomes [61]. RNA cargo and exosome membranes were monitored with multiplex reporters that were engineered by fusion of an enhanced green fluorescence protein and a tandem dimer Tomato [62]. CD63, one of the tetraspanin markers of exosomes tagged with the green fluorescent protein, can also be used for imaging of exosomes in an orthotopic nude-mouse model [63].

### 4.3. Radiolabeling of Exosomes

Even though fluorescence labeling is a convenient tool, labeling with radioisotopes enables more quantitative detection of exosomes in deep organs. A method for circumventing the problem of a decreased luminescent signal from gLuc-lactadherin in vivo was found by binding ^125^I-labeled biotin derivatives and exosomes from B16BL6 cells transfected with a streptavidin–lactadherin plasmid, which enabled dual imaging [64]. Tissue distribution of exosomes was quantitatively evaluated by measuring the radioactivity with a gamma counter.

Labeling of erythrocyte-derived exosomes using a ^99m^Tc-tricarbonyl complex showed stability in mice after intravenous administration and SPECT/CT imaging [65]. Exosome-mimetic nanovesicles labeled with ^99m^Tc-hexamethylpropyleneamineoxime, an uncharged lipophilic radiotracer converted to the hydrophilic form by intracellular glutathione, were reported to show high serum stability and uptake by the liver and spleen [66]. In addition, radiolabeling of exosome membrane with ^111^In via chelation was compared with labeling using a ^111^In-tropolone complex diffused into the exosomal lumen, and was applied in biodistribution analysis in mice [67].

Labeling of exosomes with positron-emitting radioisotopes will be helpful for obtaining images with better contrast and spatial resolution by using PET [68]. ^64^Cu-chelation and PEGylation of exosomes enhance detectability and tumor retention in tumor-bearing mice [69].

## 5. Techniques for Quantitative Analysis of Exosomes

Currently, various techniques have been developed as biological assays of surface markers and internal contents of exosomes. Western blotting, enzyme-linked immunosorbent assay (ELISA), and fluorescence-activated cell sorting (FACS) by flow cytometry can be used for analysis of exosomal surface proteins [70,71,72]. For commercialization as an ELISA kit, at least a pg/mL (pM) level of sensitivity, approximately three digits of dynamic range, and a coefficient of variation below 5% are required [73]. Cytokine array, ELISA, multiplex system, liquid chromatography (LC), mass spectrometry (MS), quantitative polymerase chain reaction, miRNA array, and capillary electrophoresis are techniques for analysis of internal contents and RNA profiling of exosomes.

More than 4600 proteins, including proteins from cytosol, plasma membrane, Golgi apparatus, and endoplasmic reticulum, are associated with exosomes. They are proteins involved in membrane transport and fusion (GTPase and annexin), tetraspanin proteins (CD9, CD63, CD81, and CD82), heat shock proteins, proteins involved in in vivo synthesis of a multivesicular body (Alix and TSG101), cytoskeleton proteins (actin, tubulin, and syntenin), lipid-related proteins, and phospholipid hydrolase. Analysis of these proteins can play an important role in pharmacokinetic analysis of exosomes, as exosomes express characteristic proteins that reflect the type of the original cells.

The yield of exosomes obtained from 400 μL of serum samples is known to be 15–18 μg in terms of total protein (quantified by using the Bradford assay) and 10–15 μg in terms of total nucleic acid. When applying existing techniques such as LC-MS/MS, Western blotting, FACS by flow cytometry, and immunoelectron microscopy using immunogold labeling to detect and quantitate exosome-related proteins, it is necessary to optimize assays for pharmacokinetic studies on exosomes [74,75,76].

By using analytical method validation that scientifically proves that the probability of determination error due to errors in an analytical method falls within the acceptable range, it is possible to verify that each analytical method shows results consistent with its intended purpose as well as reproducibility and reliability. That is, parameters such as linearity for the concentration of exosomes in a certain range of samples and minimum detectable concentration (detection limit) of exosomes in the sample should be evaluated. Some techniques need pretreatment or extraction to quantify exosomes in tissues, organs, or body fluids. Background noise should also be overcome to obtain a sufficient signal-to-noise ratio [77,78].

Physical characteristics of labeled exosomes should also be checked for quality control [79]. Nanoparticle tracking analysis and tunable resistive pulse sensing allow for measurement of the diameter, surface charge, and number of exosomes [80,81]. Raman spectroscopy provides structural information non-destructively with a small amount of samples [82], and the detectability can be improved by surface-enhanced Raman spectroscopy [83]. Recently, microfluidics with several types of sensors have also been developed for analysis of exosomes [84,85,86].

## 6. Conclusions

Recently, there has been an explosive rise in studies of the clinical application of exosomes. Molecular imaging accompanied by labeling techniques provides an ADME profile of exosome-based therapy. Recent analytical methods and pharmacokinetic modeling can be used for quantifying exosome concentration in tissue or blood samples and calculating pharmacokinetic parameters. At this point, analytical standard guidelines on pharmacokinetic evaluation of exosomes require urgent attention. It will enable us to predict the therapeutic efficacy and toxicity of exosomes in a clinical setting.

## Figures and Tables

**Table 1:** Pharmacokinetic parameters calculated according to studies on absorption, distribution, metabolism, and excretion of exosomes.

Experimental Model	Pharmacokinetic Parameters	Ref
Fluorophore-exosomes (10 nmol, i.v.) (CD-1 mice)	t_1/2_α = 6.85–8.79 min, t_1/2_β = 149–792 min, k_el_ = 0.0003–0.0052 min^−1^, AUC = 5922–25759%ID⋅g^−1^⋅min^−1^), CL = 0.0054–0.0154 mL⋅min^−1^, V_d_ = 2.4321–8.4773 mL	[22]
gLuc-lactadherin-exosomes (5 μg, i.v.) (macrophage-depleted mice)	AUC = 160 ± 33% of ID⋅h/mL, MRT = 3.57 ± 0.68 h, CL = 0.651 ± 0.157 mL/h	[23]
Milk exosomes (transport kinetics in Caco-2 and IEC-6 cells)	K_m_ = 55.5 ± 48.6 μg/200 μL (Caco-2), K_m_ = 152 ± 39.5 μg/200 μL (IEC-6)	[25]
^125^I-exosomes (4 × 10^5^ cpm, i.v.) (Parkinson’s disease mouse model)	CL = 0.016 mL/min, V_ss_ = 3.15 mL, MRT_last_ = 0.91 h, MRT_inf_ = 3.40 h	[26]

i.v. = intravenously; t_1/2_α = half-life during distribution; t_1/2_β = half-life during elimination; k_el_ = elimination rate constant; AUC = area under the concentration time curve; CL = clearance; V_d_ = volume of distribution; gLuc = gaussia luciferase; MRT = mean residence time; K_m_ = Michaelis constant; cpm = counts per minute; V_ss_ = volume of distribution at steady state; MRT_last_ = mean residence time from 0 h to the time of the last positive concentration; MRT_inf_ = mean residence time extrapolated to infinity.
